# Kearns–Sayre Syndrome Minus: Two Cases of Identical Large-Scale Mitochondrial DNA Deletions with Presentations outside the Classical Triad

**DOI:** 10.1155/2022/4153357

**Published:** 2022-04-23

**Authors:** Shir Wey Gloria Pang, Hencher Han Chih Lee, Carol Ng Wing kei, Eric Kin Cheong Yau, Joannie Hui

**Affiliations:** ^1^Department of Paediatrics and Adolescent Medicine, Hong Kong Children's Hospital, Kowloon Bay, Hong Kong; ^2^Department of Pathology, Princess Margaret Hospital, Kwai Chung, Hong Kong; ^3^Department of Radiology, Hong Kong Children's Hospital, Kowloon Bay, Hong Kong; ^4^Department of Paediatrics and Adolescent Medicine, Princess Margaret Hospital, Kwai Chung, Hong Kong

## Abstract

A curious triad of retinitis pigmentosa, external ophthalmoplegia, and complete heart block was presented by Sayre et al. in 1958. Since then, the disorder named Kearns–Sayre syndrome (KSS) has come to represent patients with mitochondrial DNA deletions presenting before adulthood, primarily with chronic progressive external ophthalmoplegia (CPEO) and pigmentary retinopathy. However, it is increasingly noted that the presentations can well be variable despite similar genetic deletions. Here, we present two cases with identical large-scale mitochondrial DNA deletions but very dissimilar outlook.

## 1. Introduction

Mitochondria exist in every cell except for mature erythrocytes. They function as the energy convertor factory of the cell. When the mitochondria fail, energy crises in the cells occur resulting in dysfunction of different organs. Mitochondrial cytopathies resulting from single large-scale deletions of mitochondria DNA include Kearns–Sayre syndrome (KSS), Pearson marrow-pancreas Syndrome (PMS), and chronic progressive external ophthalmoplegia (CPEO). KSS was first described in Mayo Clinic in 1958 [1].

Classically, KSS has a triad of features, including the presence of progressive external ophthalmoplegia, pigmentary retinopathy, and an age of onset younger than 20 years. Additionally, one or more of the following features: (1) heart block, (2) cerebellar ataxia, or (3) increased cerebrospinal fluid (CSF) protein level (>100 g/L) must be present for the diagnosis of KSS. Patients who, in addition to PEO, present with some systemic features but not yet fulfilling the definition of KSS, are regarded as either “KSS minus” or “PEO plus” [2]. In addition to clinical features, muscle biopsy or genetic testing is now routine for definitive diagnosis [3].

Here, we report two cases of genetically proven KSS. One case presented hypoglycaemia and urine profiling, whilst the other with multiple endocrinopathies.

### 1.1. Case 1

CC is the first born child to nonconsanguineous Chinese parents. He weighed 3.6 kg at birth. Antenatal and perinatal history was unremarkable.

He experienced two episodes of symptomatic hypoglycaemia during childhood. The first episode occurred when he was 5 years old. Having previously been well, he was noted one morning to be in a drowsy and sweaty state. He was hypoglycaemic with dextrostix of 2 mmol/L. Urine ketone was not available at that juncture. There was mild metabolic acidosis, but serum lactate, ammonia, total carnitine, and acylcarnitine profiles were normal. Urine metabolic screening revealed moderate generalised aminoaciduria, lactic acid, 3-hydroxy propionic, 3-hydroxy isovaleric acid, and tiglylglycine, which was compatible with multiple carboxylase deficiency.

He experienced a second episode of hypoglycaemia at 6 years of age. He had been suffering from repeated vomiting and was found hypoglycaemic upon attendance to Accident and Emergency Department. Blood glucose was 1.9 mmol/L, and there was associated metabolic acidosis. Urine ketones were strongly positive. Ammonia, lactate, and pyruvate were normal. The patient then underwent further investigations including a prolonged fasting for over 8 hours, but all investigations came back normal. Blood sugar level was 4.8 mmol/L after an overnight fast. Urine metabolic screening was repeated but did not demonstrate significant abnormalities. Subsequently, CC suffered from repeated vomiting on several occasions, but serial urine specimens were normal. The working diagnosis was cyclical vomiting syndrome.

**Figure 1:**
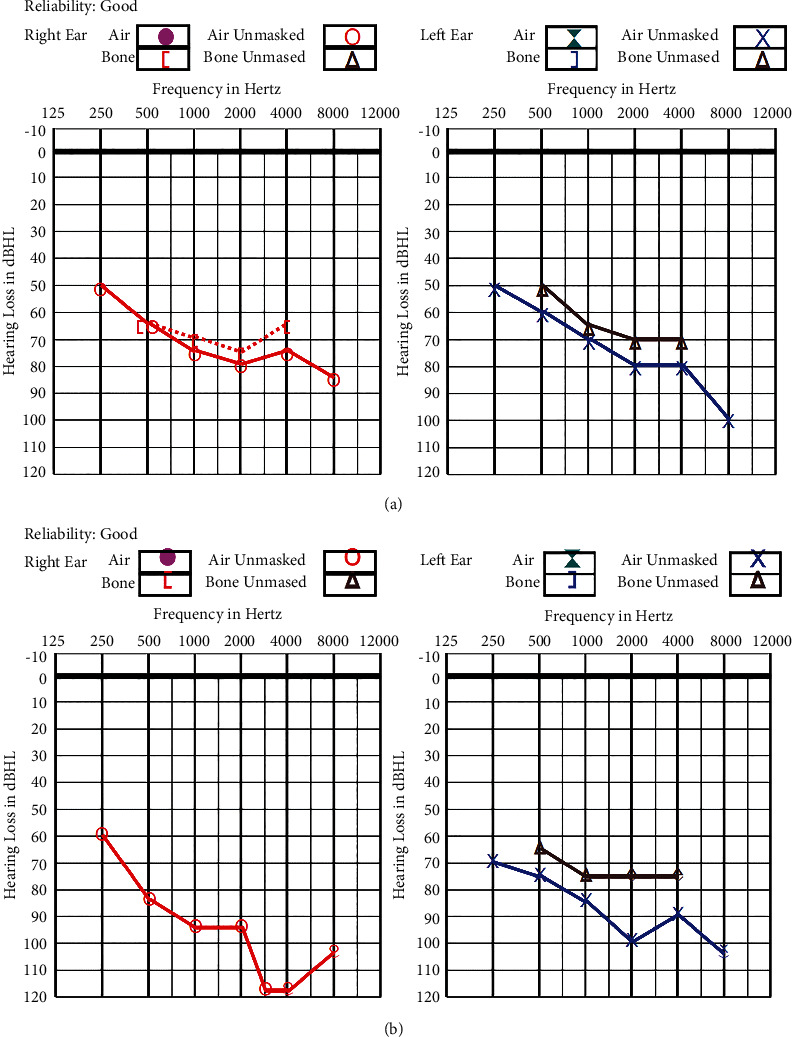
(a) Audiogram at presentation at 12 years of age. Bilateral severe sensorineural hearing loss. (b) Audiogram at 22 years of age. Profound sensorineural hearing loss over right ear and severe hearing loss over left ear.

CC later developed hearing difficulties. He was diagnosed to have bilateral sensorineural hearing loss at the age of 7 and required hearing aids.  Figure 1 shows his audiogram in earlier years and subsequent deterioration.

CC's height had been satisfactory in the early years of life, with his height lying in the 10–25th centile up till 5 years of age. His weight was all along in the 3rd to 10th centile. This was partially attributed to the fact that he was a small eater and was easily full. At 6 years of age, both his height and weight dropped to the 3rd centile; by 12 years of age, he was 12 cm below the 3rd centile. Serial bone age revealed a delay of approximately 1.5 years from the chronological age. His parents are of moderate build, with a midparental height at the 25th centile.

He started to develop eye abnormalities since the age of 15, which progressed to severe ptosis with restricted horizontal and upward gaze. He adopted an abnormal chin up posture to compensate for the severe ptosis. His lenses were mildly hazy, but his visual acuity remained normal. Fundoscopy also revealed a normal retina.

Peripheral blood was taken for genetic studies which confirmed heteroplasmic m.8649_16084del, related to KSS and Pearson marrow-pancreas syndrome.

After the diagnosis was made, he underwent yearly electrocardiography (ECG) and echocardiography assessment and these have so far been normal. Currently, he is on folinic acid and ubiquinol.

### 1.2. Case 2

**Figure 2:**
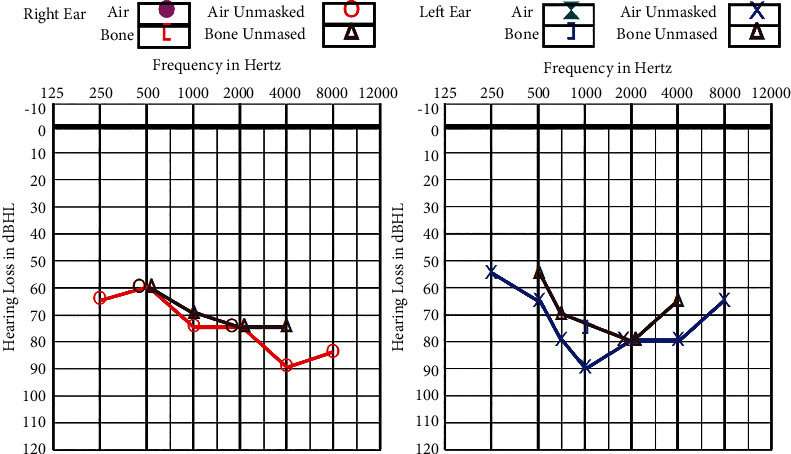
Audiogram at presentation at 6 years of age. Bilateral severe sensorineural hearing loss at 60–90 dB.

WH is an only child to a nonconsanguineous Chinese family. She first presented at the age of 7 with bilateral sensorineural hearing loss. Pure tone audiometry at that juncture showed moderate sensorineural hearing loss of 40 dB, and she was prescribed hearing aids soon after diagnosis. Subsequently, her hearing deteriorated, resulting in profound hearing loss of 80 dB 4 years later.  Figure 2 shows her audiogram.

She also suffered from dizziness and nonpulsatile tinnitus with workup attempted but posturography test was inconclusive as the patient was uncooperative.

**Figure 3:**
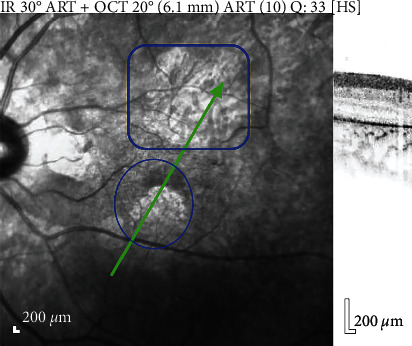
Optic coherence tomography scan showing a patch of atrophy in the outer retina inferotemporal to the fovea with photoreceptor layer disruption.

Her night vision began to falter at around the same time. Examination of the fundi revealed pigmentary changes. Electroretinography demonstrated decreased *b* wave amplitude in both rods and combined responses in scotopic responses. There was also decreased *a* and *b* wave amplitude and 30 Hz flicker responses in photopic responses. The findings were compatible with rod cone dystrophy.  Figure 3 shows her optic coherence tomography scan.

Her weight and height had been along the 3rd centile up till the age of 8 years. Both dwindled subsequently, and her weight was 4 kg below 3rd centile and height 10 cm below 3rd centile by the age of 10. She was tested for Turner's syndrome which was negative.

Later that year in clinic, she complained of polyuria, polydipsia, and nocturia. Blood glucose was 32.8 mmol/L with accompanying ketosis and compensated metabolic acidosis. The serum pH was 7.39, bicarbonate was 15.9 mmol/L, and base excess was down to −8.1 mEq/L. She was diagnosed with diabetic ketoacidosis, and after stabilisation has been maintained on four insulin injections. Anti-islet cell antibodies were negative.

In view of the diabetes mellitus, hearing loss, and visual problems, she was tested for Wolfram syndrome and was negative.

By the age of 12 years, she developed hypocalcaemia and hypoparathyroidism with serum calcium of 1.78 mmol/L, ionised calcium 0.93 mmoL/L, serum phosphate 2.23 mmol/L, and parathyroid hormone 0.56 pmol/L (normal 1.3–9.3 pmol/L). She was started on calcium carbonate 1000 mg daily and vitamin D supplementation in the form of calcitriol 0.25 mcg daily. Computed tomography of the brain revealed calcifications at the left temporal lobe, bilateral basal ganglia, and cerebellum.

Because of multiple endocrine problems, she underwent autoimmune screening. Antiadrenal antibodies, antithyroid stimulating hormone receptor antibodies, and anti-human tissue transglutaminase antibodies were all negative. Her thyroid, adrenal, and gonadotrophin axes remained intact. Her progression through puberty had been uneventful and menarche occurred when she was 15 years of age.

**Figure 4:**
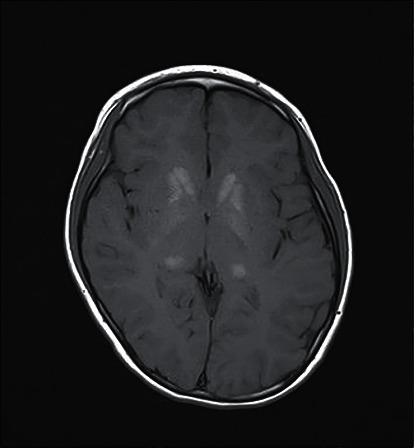
MRI brain showing T1 hyperintensities at caudate head, putamen, and thalamus caused by calcium.

**Figure 5:**
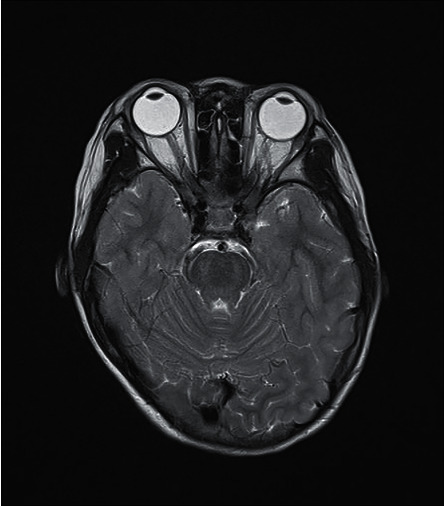
MRI brain showing T2 hyperintensities at posterior and bilateral pons.

Neurologically, she had normal muscle tone and power, though she did complain of lack of energy. Her gait was coordinated, and she had no cerebellar signs. Magnetic resonance imaging of brain showed T2 hyperintensities in bilateral anterior globi pallidi, midbrain, and cerebral peduncle. Figures 4 and 5 show her MRI abnormalities.

Genetic study with peripheral blood revealed heteroplasmic deletion at m.8649_16084del. WH continued to have half yearly assessment for her cardiac function which has remained normal.

## 2. Discussion

**Figure 6:**
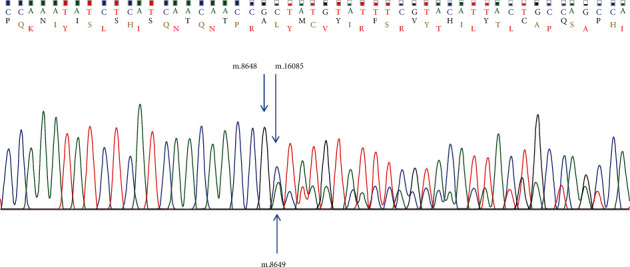
DNA sequencing electrophoretogram demonstrating the breakpoint. Blue arrows from the top indicate the nucleotides on the two sides of the breakpoint (m.8648 and m.16085). Another blue arrow from the bottom indicates the nucleotide m.8649 in the wild type.

Both patients harboured the same heteroplasmic m.8649_16084del mutation related to CPEO, KSS, and PMS syndrome. It is the second most common large-scale mitochondrial DNA deletion amongst those previously described and consists of 8/13 genes encoding for mitochondrial OXPHOS subunits and 8/22 tRNAs [4]. The genetic testing was performed on peripheral blood samples, with long-range polymerase chain reaction amplification and direct sequencing of the breakpoints performed.  Figure 6 shows the DNA sequencing electrophoretogram.

The 7436-bp large mtDNA deletion was previously described as a common recurrent variant with a flanking 12-nucleotide direct repeat of CATCAACAACCG at each end in the *MT-ATP6* gene and the D-loop region, respectively, encompassing mainly genes encoding components of respiratory complexes I, III, and IV, in addition to multiple transfer RNA. It was first demonstrated in cardiomyocytes of three patients who passed away in middle ages with cardiomyopathies [5]. The deletion was also detected in hippocampectomy specimens with hippocampal sclerosis [6], and mtDNA of infertile sperm [7, 8], muscle biopsies from patients with liver cirrhosis and end-stage renal failure [9, 10], and hair follicles from aged individuals [11]. The deletion was also detected in patients with PMS and chronic fatigue syndrome [12, 13], and together with a *OCA2* variant in a patient with albinism and KSS phenotype [14].

PMS presents in infancy, and the diagnosis is made in the presence/absence of anaemia and/or vacuoles in erythroblasts or other haematologic cell lineages and pancreatic dysfunction. Neither of our patients have documented anaemia nor haematological abnormalities.

CPEO is a muscle-specific disease affecting the eye. KSS is genotypically a continuum of CPEO, with a classical triad of progressive external ophthalmoplegia, pigmentary retinopathy, and an age of onset younger than 20 years. KSS has longer deletions and larger number of deleted tRNAs than CPEO. It is probable that patients with longer deletions and more deleted tRNAs induce the development of KSS phenotype or enhance multisystemic involvement [15].

Both our patients fulfill the criteria of young onset of symptoms for KSS. However, our first patient does not suffer from pigmentary retinopathy and the second patient does not have PEO. Both of them have yet to fulfil the additional criteria of cerebellar symptoms, complete heart block, or high cerebrospinal fluid protein content due to white matter degeneration (lumbar puncture was not performed and thus could not be commented upon). Thus both are classified as PEO plus, or KSS minus cases.  Table 1 illustrates the different clinical features found in our patients.

Abnormal urine profile (and hypoglycaemia) had first brought Case 1 to the attention of metabolic paediatrician, who initially suspected him to have multiple carboxylase deficiency. A recent paper analyses abnormal urinary organic acids in patients with single mitochondrial DNA deletions. Semeraro reported that metabolites including lactate, 3-hydroxybyturate, 3-hydroxyisobutyrate, fumarate, pyruvate, and 2-hydroxybutyrate, 2-methyl 2,3-dihydroxybutyrate, 2-ethyl 3-hydroxypropionate, 3-methylglutaconate, and tiglylglycine appeared in the majority of urinary samples saved from the eight patients with PMS [16]. Patients with KSS tended to have normal urine organic acid profiles, and if abnormal, the metabolites were similar to those found in PMS but were less pronounced. Our patient also presented with tiglylglycine which was noted in that paper in PMS urine patient samples.

Case 2 presented to the paediatric team with short stature, followed by diabetes mellitus and hypoparathyroidism. Hypoparathyroidism occurs in 7–8% of KSS patients. The cause of hypoparathyroidism is not fully understood, though it is known that PTH is produced in mitochondrial packed clear and oxyphil cells. Low ionised calcium concentrations is detected by the calcium sensing receptor, which activates protein kinase C and G proteins, coupling with adenylate cyclase to produce ATP. In the case of mitochondrial failure, it is postulated that PTH production and calcium release would be hindered [17].

Diabetes mellitus in mitochondrial disease can be both insulinopenic and insulin-resistant in nature. Glucose stimulates insulin via the closure of ATP-driven potassium channels in the cell membrane. In mitochondrial patients, impaired oxidative phosphorylation and decreased ATP production leads to insulin secretion failure [18]. Diabetes can also result from insulin resistance, as defects in mitochondrial metabolism in skeletal muscles can lead to reduced fat oxidation capacity and abnormal lipid accumulation by reducing glucose intake [19].

The association of diabetes with hypoparathyroidism has frequently been described in KSS, and postulations have been made as to why they occur together. Isotani postulates that genetic linkage and mitochondrial dysfunction likely play a role. In a paper reporting a case of polyglandular autoimmune syndrome (PGA) Type 1, they simultaneously reported four Japanese patients with positive HLA-A24 and CW3 antigen was also present in presenting with IDDM and hypoparathyroidism [20]. Antibodies to substantiate autoimmunity for IDDM and hypoparathyroidism in KSS have been scarce. Isotani's case of IDDM and hypoparathyroidism was negative for ICA and antiparathyroid antibodies [20], and Wilichowski reported a similar case where negative antipancreatic tissue, and antiparathyroid antibodies were undetectable [17]. In a few KSS cases involving diabetes, low titres of ICA had been described [21], though in most cases, they have been undetectable. In another case of KSS with growth hormone deficiency, IDDM, and hypothyroidism, antithyroidal antithyroglobulin and anti-thyreoperoxidase antibodies were positive. In the latter case, the authors postulate that the OXPHOS deficiency could have caused a thyroid cell injury leading to auto antigen production and the presence of antibodies in KSS [15].

Our second patient had presented with diabetic ketoacidosis, with a very high HbA1c and high postprandial glucose level. She was started on multiple doses of insulin (rapid acting and long acting) right from the start. She remains insulinopenic with C-peptide of 0.15 nmol/L.

Her glucose levels are very difficult to control, as her appetite is frequently governed by concomitant disturbing gastrointestinal (GI) problems. The cause of these symptoms in mitochondrial patients is unclear but may involve loss of intestinal cells of Cajal and marked atrophy of muscular propia [22]. All in all, symptoms including anorexia, constipation, reflux, diarrhoea, and dysphagia affects up to 50% of mitochondrial patients. Anorexia and bloating sensation were the most prominent in our patient, and it means she can take hours to finish a meal. Insulin titration is very difficult. Medications for gastrointestinal symptoms for symptomatic relief have been suggested including tricyclic antidepressants for chronic irritable bowel syndrome type pain and cyclical vomiting, proton pump inhibitors and antispasmodics for reflux disease, and domperidone for constipation. Domperidone had been tried in our patient without significant improvement. Coenzyme Q10 was given empirically as for other mitochondrial disorders, in the hope of some improvement of GI symptoms, but this was futile. She was started on dimenhydrinate by a private practitioner for nausea, and she reported significant improvement on symptoms. However, due to possible effects of prolonged QT interval in a patient at risk of cardiac conduction abnormalities, the medication was terminated.

Cardiac manifestations remain the most sinister complication of KSS and are the most important prognostic indicator. The continuous demand of ATP from oxidative metabolism renders cardiac myocytes the cells with the highest volume density of mitochondria in the body [23]. 57% of KSS patients suffer from cardiac problems, which primarily present as syncopal attacks, heart failure, and cardiac arrest due to cardiac conduction failure [24]. Pathological findings in KSS typically involve distal bundle of His as well as bundle branches and infra nodal conductions [25, 26]. ECG changes begin as prolonged PR interval and progress over time to 2nd and 3rd degree heart block. The definitive treatment for presence of atrioventricular (AV) blockage is permanent pacemaker (PPM) implantation. The 2012 guidance for device-based therapy of cardiac rhythm abnormalities published by American College of Cardiology Foundation and American Heart Association and Heart Rhythm Society recommends PPM implantation (class 1 level evidence B) for KSS patients with third degree and advanced second degree AV block at any anatomic level whether or not it is associated with symptoms set (class 1 level of evidence: B). For patients with any degree of AV block (including first degree AV block) or any fascicular block with or without symptoms, PPM may still be recommended (class IIb indication) because there may be unpredictable progression of AV conduction disease (level of evidence: B) [27]. The 2007 European Society of Cardiology guidelines for cardiac pacing recommended PPM implantation for patients with neuromuscular disease with any degree of vascular block (Class IIA, level of evidence C) or with second or third degree AV block (class I level of evidence B1) [28].

In the largest series of KSS to date with 35 patients, Khambatta noted that all four deaths in their series were sudden cardiac death cases, of which three had not presented with ECG changes prior to the event and were thus not given PPM/implantable cardioverter defibrillator. Only two of these cases had previous cardiac symptoms, one had syncope and the other with history of cardiac arrest and ventricular arrhythmia. Amongst these four death cases, one patient, who was diagnosed by mtDNA, did not have PEO; in another patient diagnosed by the muscle biopsy, there was no pigmentary retinopathy. Thus, two out of the four death cases were indeed KSS minus patients [29]. These findings are of interest to our patients even though neither of our patients has been found to have arrhythmia so far, because being KSS minus patients does not equate having lesser risk to develop dire comorbidities. Indeed, it reflects that be it KSS minus or not, current guidelines may not be adequate to screen out all vulnerable subjects at risk of sudden death.

## 3. Conclusion

KSS is a multisystemic disease and presents with a multitude of symptoms even if the genetic deletion is similar. The presentations first discussed in 1958 have been well accepted. As research progresses, many are reporting similar symptoms outside the initial criteria set, including short stature, deafness, and endocrinopathies. Large-scale genetic deletions known to cause KSS are being confirmed, and mechanisms of mitochondrial dysfunction leading to such conditions are being investigated. Thus, in the near future, the criteria of KSS may be accustomed to fit these cases which fundamentally have the same genetic diagnosis and should be monitored for cardiac risks during their lifetime.

## Figures and Tables

**Table 1:** Clinical and laboratory features of the two patients with Kearns–Sayre syndrome.

Affected systems	Features	Case 1	Case 2
Clinical features			
Growth	Short stature	−2.8 SD for height	−2.6 SD for height
	Failure to thrive	−2.5 SD for weight	−2.5 SD for weight

Audiology	Sensorineural hearing loss	Severe to profound	Severe

Endocrinology	Abnormal glucose status	Ketotic hypoglycaemia with glucose 1.9 mmol/L	Diabetic ketoacidosis with glucose 32.8 mmol/L
	Hypoparathyroidism	Not affected	PTH 0.56 pmol/L (1.3–9.3pmol/L), iCa 0.93 (1.2–1.4 mmol/L), Ca 1.6 mmol/L (2.1–2.5 mmol/L), PO4 1.78 mmoL/L (1.12–1.45 mmol/L)

Ophthalmology	Retinopathy	Not affected	Pigmentary retinopathy, retinal atrophy
	Ophthalmoplegia	Progressive external ophthalmoplegia	Not affected

Musculoskeleletal	Eye muscles	Ptosis	Not affected
	Skeletal muscles	Not affected	Generalised muscle weakness
Laboratory features			

Urine x metabolic screening		Generalised aminoaciduria, lactic acid, 3-hydroxy proprionic, 3-hydroxyisovaleric acid, and tiglylglycine	No abnormalities

Lactate	Raised lactate	2.6–8.9 mmol/L	Not raised

## Data Availability

No data are available.
